# In-depth analysis of chloride treatments for thin-film CdTe solar cells

**DOI:** 10.1038/ncomms13231

**Published:** 2016-10-24

**Authors:** J. D. Major, M. Al Turkestani, L. Bowen, M. Brossard, C. Li, P. Lagoudakis, S. J. Pennycook, L. J. Phillips, R. E. Treharne, K. Durose

**Affiliations:** 1 Stephenson Institute for Renewable Energy and Department of Physics, University of Liverpool, Liverpool L69 7ZF, UK; 2 Department of Physics, Umm Al-Qura University, KSA, Mecca Al Taif Road, Mecca 24382, Saudi Arabia; 3 Department of Physics, G.J. Russell Microscopy Facility, Durham University, South Road, Durham DH1 3LE, UK; 4 University of Southampton, School of Physics & Astronomy, Southampton, Hants SO17 1BJ, UK; 5 Centre for Photonics and Quantum Materials, Skolkovo Institute of Science and Technology, Moscow 143026, Russia; 6 Materials Science and Technology Division, Oak Ridge National Laboratory, 1 Bethel Valley Rd, Oak Ridge, Tennessee 37831, USA; 7 Department of Materials Science and Engineering, National University of Singapore, Singapore 117576, Singapore

## Abstract

CdTe thin-film solar cells are now the main industrially established alternative to silicon-based photovoltaics. These cells remain reliant on the so-called chloride activation step in order to achieve high conversion efficiencies. Here, by comparison of effective and ineffective chloride treatments, we show the main role of the chloride process to be the modification of grain boundaries through chlorine accumulation, which leads an increase in the carrier lifetime. It is also demonstrated that while improvements in fill factor and short circuit current may be achieved through use of the ineffective chlorides, or indeed simple air annealing, voltage improvement is linked directly to chlorine incorporation at the grain boundaries. This suggests that focus on improved or more controlled grain boundary treatments may provide a route to achieving higher cell voltages and thus efficiencies.

## Abstract (web summary)

High performance CdTe thin film solar cells typically require a chloride activation treatment. Here, Major *et al*. show that the main effect of the most effective chloride-based treatments is chloride accumulation at grain boundaries and that it results in improved open circuit voltages.

Cadmium telluride thin-film solar cells have seen a rapid increase in efficiency in the past 4 years, with laboratory scale devices increasing from 16.7% in January 2012 (ref. 1) to 21.5% in June 2015 (ref. 2) while CdTe module efficiencies have now surpassed that achievable with multi-crystalline silicon^2^. To achieve such high efficiencies in CdTe solar cells the dominant process step remains the chloride ‘activation' treatment, wherein chlorine is diffused into the CdTe layer by annealing after exposure to CdCl_2_ or MgCl_2_ (ref. 3). Without such treatment, the cell efficiency is typically <5%. Despite the level of technological advancement CdTe cells have now reached, the chloride activation process is still not fully understood. The traditional approach to investigating the impact of the chloride treatment has been to compare CdCl_2_-treated and -untreated devices and to analyse the metallurgical and electrical changes. There are many papers that follow this approach and while it is not without merit, it is also inherently limited. Such a wide array of changes occur during treatment; p-type doping^4^, grain recrystallization^5^, intermixing between the CdTe/CdS layers^6^, junction formation^7^, modification of grain boundary carrier collection^8^; that comparison with as-grown (that is, untreated) devices, which are always low performance, makes it difficult to isolate the key changes. Our previous development of the MgCl_2_ treatment^3^ allows us now to take a different approach: we are able to compare two chloride treatments that we know to yield high efficiency, namely CdCl_2_ and MgCl_2_, with two which yield some performance improvements but not to the same extent, these being NaCl and KCl, as well as untreated devices. This allows us to develop a more detailed understanding of exactly why compounds such as MgCl_2_ are effective, whereas compounds such as NaCl are not. Comparisons are also able to be made with air annealing, but use of the ineffective chlorides offers an important like-for-like comparison where post-growth processing conditions are kept as similar as possible. By making these comparisons it allows us to separate the different impacts the chloride treatment has and to identify the key roles that the chlorine has in isolation from the effects of annealing.

In order to study the impact of the chloride treatment in isolation as much as possible, we have sought to minimize the influence of other secondary dopant species oxygen and copper. For the highest efficiencies to be achieved, we would typically include an oxidized nanostructured CdS layer (CdS:O) and in-diffuse copper into the CdTe back surface^3^. Both of these are known to improve performance, but are liable to mask the impact of the chloride treatment and were thus removed from the cell fabrication process. As a result, peak cell efficiencies are lower than the maxima achievable, however, their removal affords a cleaner comparison of the chloride treatments. The aim of this work is placed on comparative analysis rather than peak efficiency.

This work reports comparative in-depth materials and device characterization of CdTe solar cells with these different treatments. Through this analysis, we demonstrate that effective chloride treatments improve the cell *V*_OC_ via a mechanism of chlorine incorporation at the grain boundaries, which increases the minority carrier lifetime and improves the junction carrier transport. This work presents a comprehensive comparative study of MgCl_2_ (as well as NaCl and KCl)-treated CdTe solar cells with the more standard CdCl_2_ treatment.

## Results

### Cell performance for varied annealing conditions

We have previously reported on the variation in optimized device efficiency resulting from various chloride treatments^3^,^9^. For reference, efficiency data is listed in Supplementary Table 1.

To summarize, all chloride treatments tested generate some increase in performance in comparison to the as-grown (untreated) sample but CdCl_2_ and MgCl_2_ treatments produce markedly higher efficiencies than the others. In terms of device performance, we consider these two treatments to be essentially equivalent. However, since NaCl and KCl treatments impart some improvement to device performance, it is tempting to say that they are all ‘effective' to an extent. Further and more in-depth comparison of the change in individual device performance parameters from current-voltage (*JV*) analysis for these different treatments as a function of annealing conditions suggests differently though.

**Figure 1:**
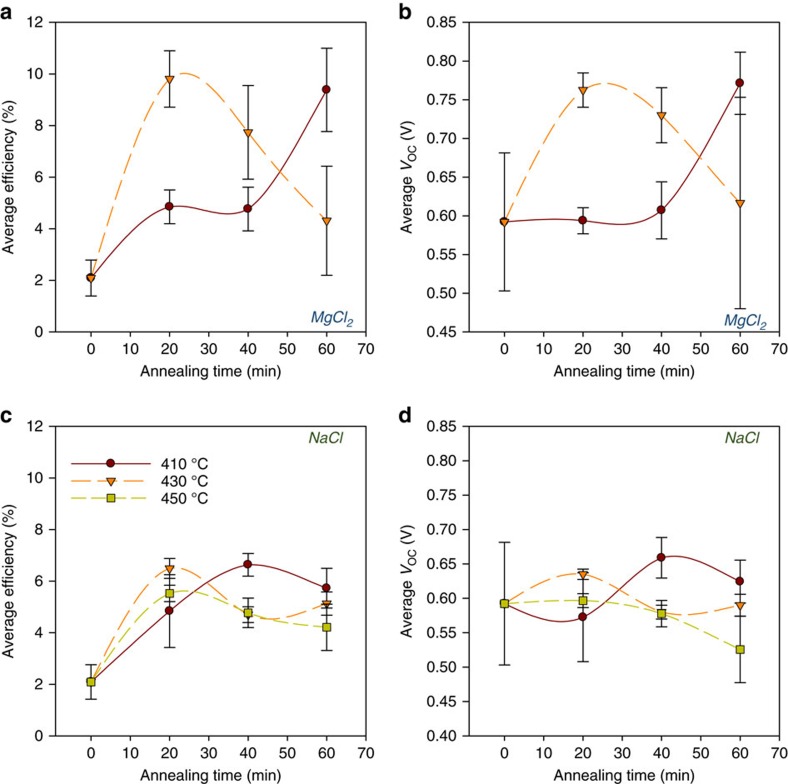
Cell performance for various MgCl_2_ and NaCl treatment conditions. Variation in average performance parameters extracted from *JV* data for MgCl_2_ and NaCl treatments performed for a range of treatment times and temperatures 410 °C (red), 430 °C (orange) and 450 °C (yellow). (**a**) MgCl_2_—efficiency (**b**) MgCl_2_—open circuit voltage, (**c**) NaCl—efficiency and (**d**) NaCl—open circuit voltage. Data for treatment at 450 °C using MgCl_2_ is not shown since cells treated at that temperature delaminated. Each data point is an average of nine contacts with error bars being the standard deviation and an error term for contact size variation.

Figure 1 shows average efficiency and open circuit voltage values (*V*_OC_) determined for cells treated with either MgCl_2_, an effective treatment, or NaCl, an ineffective treatment. Each point represents the average for a cell plate with nine contacts. For each treatment, the annealing step for an individual cell plate was varied in the ranges 410–450 °C and 20–60 min, with 0 min being the equivalent as-grown (untreated) cell performance. For an untreated cell, the peak efficiency is 2.9% but with NaCl treatment this increases to a peak of 7.4%. All the performance gain is, however, via short circuit current (*J*_SC_) and fill factor increases (Supplementary Figs 1 and 2) compared with their as-grown levels. As Fig. 1 demonstrates, the *V*_OC_ shows a negligible increase, having a maximum of 670 mV for the as-grown samples and a peak of only 680 mV for the NaCl devices under any treatment condition. Indeed for a number of NaCl treatment conditions, the *V*_OC_ is in fact decreased, particularly for treatment at 450 °C. In contrast, the MgCl_2_-treated cells show a strong *V*_OC_ improvement to >820 mV associated with the large efficiency increase. It is this voltage increase rather than increases in fill factor or *J*_SC_ that distinguishes ‘effective' and ‘ineffective' chloride treatments. Hence the effective treatments, MgCl_2_ and CdCl_2_, are capable of increasing the *V*_OC_ to levels in excess of 800 mV, while ineffective treatments studied (that is, NaCl, KCl) do not exceed 700 mV irrespective of treatment conditions and indeed often lead to an active reduction in the *V*_OC_. Following these observations, it is important to establish two factors: why these ineffective treatments fail to generate a *V*_OC_ improvement and the mechanism by which the effective treatments do improve the *V*_OC_.

### Chemical composition analysis

**Figure 2:**
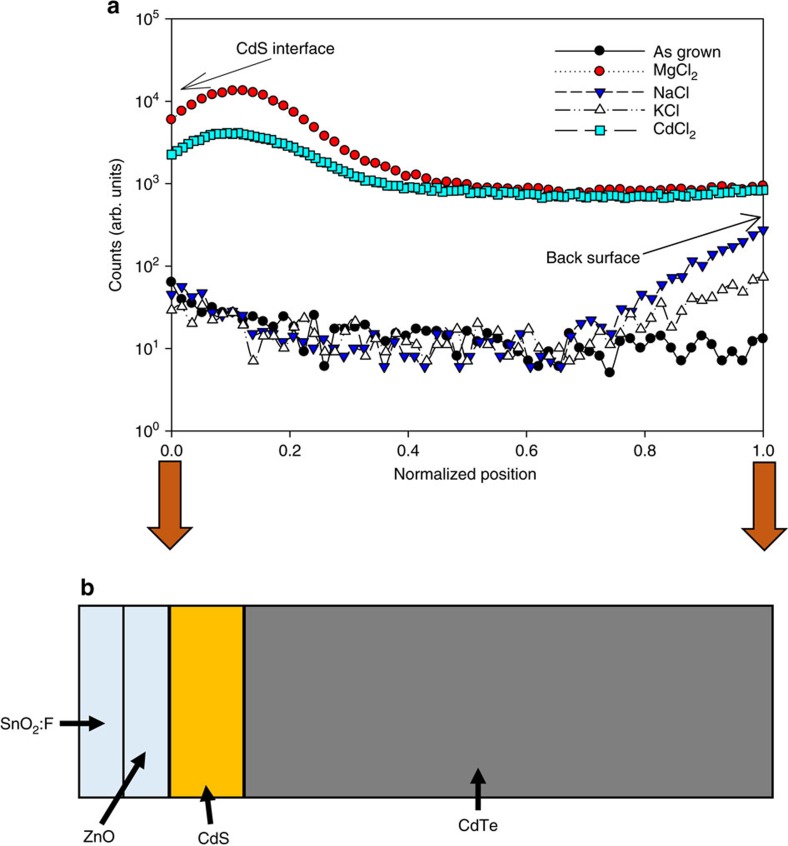
SIMS analysis of chlorine diffusion. (**a**) SIMS profiles of chlorine content as-grown (black), MgCl_2_ (red), CdCl_2_ (turquoise), KCl (white) and NaCl (blue)-treated CdTe solar cells with (**b**) schematic showing the respective position of measurement data within CdTe cell structure.

The failure of the infective treatments may be better understood by the use of secondary ion mass spectrometry (SIMS) analysis. Here we compare five samples: as-grown, MgCl_2_-treated (‘effective'), CdCl_2_-treated (‘effective'), NaCl-treated (‘ineffective') and KCl-treated (‘ineffective') cells. Figure 2 shows the SIMS chlorine profiles for the four samples with the position being normalized such that 0 is the ZnO/CdS interface (determined from sulfur profiles) and 1 is the CdTe back surface with the CdTe thickness being ∼4 μm for all cells. The SIMS profiles were obtained from all of the samples using identical instrumental conditions. Hence the measured signal in counts is directly proportional to the elemental concentration is all cases, and direct comparison of the relative chlorine concentration and relative distribution are possible. From the profiles it is clearly seen that annealing in the presence of MgCl_2_ and CdCl_2_ has led to an increase in the chlorine content of the cell of approximately two orders of magnitude compared with the background levels in the as-grown device. There is also an increase in the chlorine counts within the CdS compared with the CdTe, as is often observed for CdCl_2_-treated cells^10^. This is at least in part due to the change in matrix upon sputtering from the CdTe into the CdS: the sputter yield of chlorine from the two materials is different and this acts to increase the count rate from CdS^11^ rather than indicating a genuine increase in concentration. In contrast, both the NaCl and KCl treatments give only a minor increase in the chlorine concentration, and even then this is confined to the CdTe nearest to the diffusion surface—deeper in the CdTe, the chloride level does not rise above its background value. This is strongly indicative that the poor performance of these treatments results from an inability of NaCl and KCl to effectively introduce chlorine into the CdTe, in contrast to the case for CdCl_2_ and MgCl_2_ treatments, possibly due to the higher dissociation energies of the compounds^12^. In essence, NaCl and KCl treatments have improved the cell performance over as-grown devices in spite of little chlorine being incorporated. However, when one considers that similar increases in *J*_SC_ and fill factor to those obtained from NaCl and KCl treatment have previously been obtained by simple thermal treatment^13^ (that is, with no chloride present) the result is less surprising. Indeed direct comparison with air annealed cells (see Supplementary Fig. 3) shows a similar response to annealing conditions as for that with the ineffective chlorides present. Utilizing the same treatment ranges (410–450 °C, 20–60 min) gives similar increases in *J*_SC_ and fill factor but again the *V*_OC_ is never increased from the as-grown level. There are, however, a number of differences between the ineffective treatments and air annealing: X-ray diffraction analysis shows NaCl treatment causes an increase in <111> preferred orientation, while other treatments generate a decrease^12^; MnCl_2_ and KCl give efficiency levels well below those achievable with air annealing with a pronounced decrease in *V*_OC_ compared with as-grown devices (Supplementary Table 1). However, as the ineffective treatments fail to incorporate significant amounts of chlorine into the CdTe we may draw some parallels with air annealing. From this we can state that every process tested is capable of improving *J*_SC_ and fill factor, this occurs essentially in the absence of chlorine, but that improvement in *V*_OC_ is wholly dependent upon the incorporation of chlorine.

Having established that the incorporation of high levels of chlorine into the CdTe bulk was the root cause of the increase in *V*_*OC*_, we utilized scanning tunnelling electron microscopy (STEM) and electron energy loss spectroscopy (EELS) analysis to establish the spatial distribution of chlorine with respect to the CdTe grain structure following MgCl_2_ treatment. Previous work by Li *et al*.^8^,^14^ established that for CdCl_2_ treatment, chlorine was segregated at the grain boundaries, serving to pacify them. Other work by Abbas *et al*.^15^ has indicated that chlorine may accumulate at the CdS/TCO interface following CdCl_2_ treatment. Both of these potential mechanisms were investigated for MgCl_2_ treatment in the present work to identify commonalities with the CdCl_2_ process.

**Figure 3:**
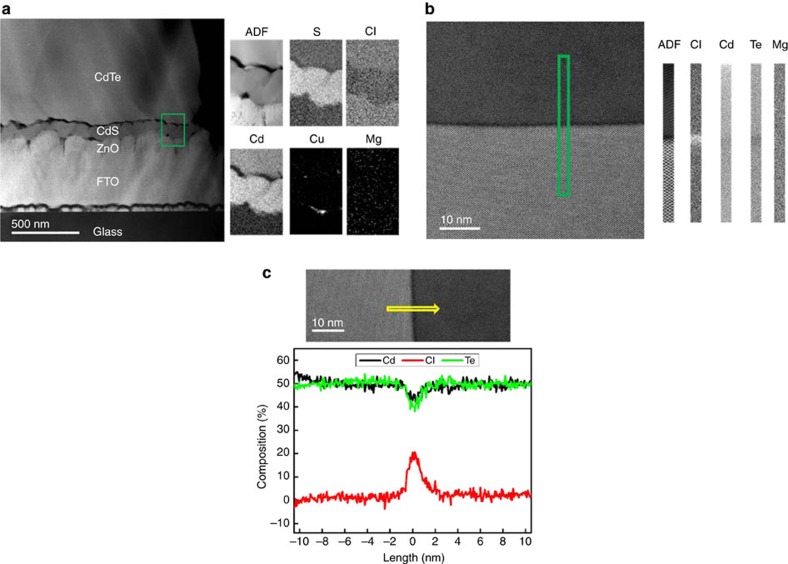
STEM and EELS analysis of MgCl_2_-treated cell. STEM annular dark field (ADF) image of a cross section of a MgCl_2_-treated CdTe solar cell, with EELS elemental maps from the region marked by a green box at (**a**) CdS/CdTe interface, (**b**) across a CdTe grain boundary and (**c**) EELS quantitative elemental profiles (from the position of the yellow arrow) for an MgCl_2_-treated cell grain boundary.

Figure 3a shows an STEM image of the FTO/ZnO/CdS/CdTe interface region for an MgCl_2_-treated cell while 3b shows a CdTe grain boundary. S, Cl, Cd, Cu and Mg maps extracted from the highlighted region via EELS analysis are also shown. There is clearly no evidence of any strong chlorine accumulation at the CdS interfaces or indeed for any magnesium accumulation at them and overall any magnesium content appears to be below the detection threshold for EELS either at the interface or grain boundaries. Only Cu shows any accumulation at the interface, with this presumed to have come from impurities in the gold back contact. Figure 3c shows a higher resolution image of a single CdTe grain boundary with the corresponding quantitative line scans of Cl, Cd and Te content across the boundary shown below. Following MgCl_2_ treatment, chlorine has accumulated at the grain boundary, with there being minimal chlorine content in the grain interiors. This matches the finding for CdCl_2_ treatment^8^ and we may, therefore, infer a generalized mechanism, namely that effective chloride treatments lead to chlorine segregation at the CdTe grain boundaries. This in turn lead to performance improvement through modification of the electrical state of the boundaries themselves, that is, the grain boundaries are in some way electrically passivated. If chlorine is not incorporated at the grain boundaries (for example, ineffective treatments or air annealing), they will remain deleterious to solar cell performance. This grain boundary accumulation is clearly, therefore, linked to the *V*_OC_, and this can be demonstrated by analysis the minority carrier lifetime.

### Minority-carrier lifetime analysis

**Figure 4:**
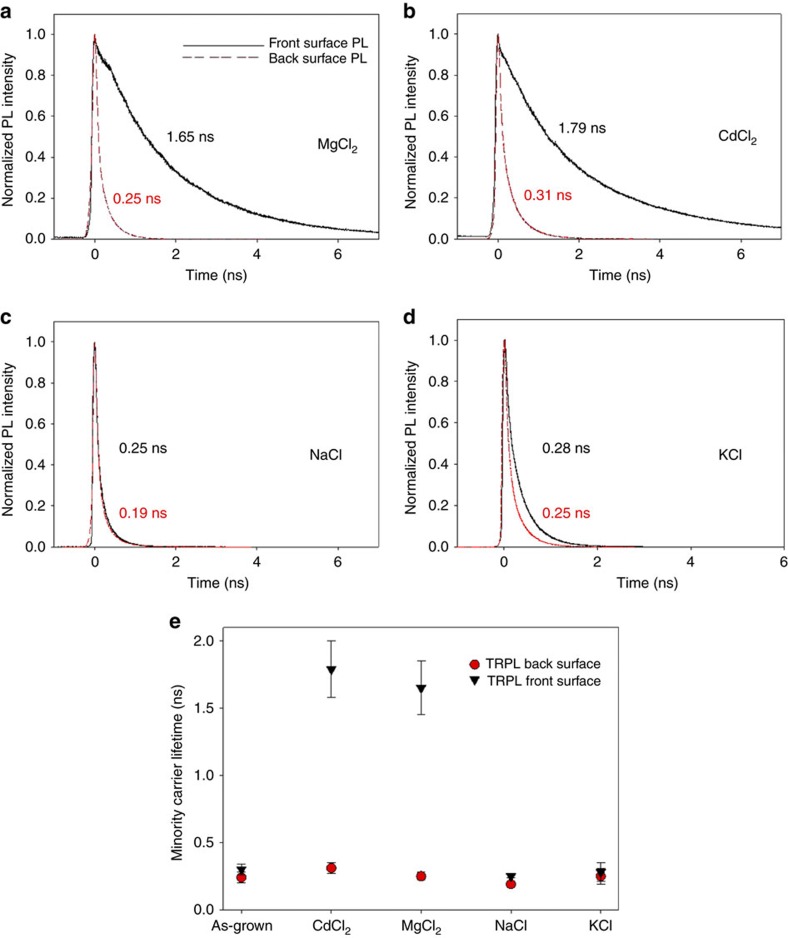
TRPL analysis of minority carrier lifetime. TRPL decay curves showing front surface (black) and back surface (red) measurements with associated minority carrier lifetimes extracted by fitting to the curve using a bi-exponential decay function (see methods section) for CdTe solar cells treated with (**a**) MgCl_2_ (lifetimes 0.25 and 1.65 ns), (**b**) CdCl_2_ (lifetimes 0.31 and 1.79 ns), (**c**) NaCl (lifetimes 0.25 and 0.19 ns), (**d**) KCl (lifetimes 0.28 and 0.25 ns) and (**e**) average carrier lifetimes (average of five measurements with error bars being one s.d. of the values) for as-grown, CdCl_2_, MgCl_2_, NaCl and KCl treatments. All measurements are shown on the same time scale to allow easy comparison, however, low carrier lifetime samples were measured for shorter time periods.

Minority-carrier lifetime has been shown to be the CdTe material property most directly correlated to cell *V*_OC_ (ref. 16). One of the primary methods used to study it is time-resolved photoluminescence (TRPL). Determination of accurate carrier lifetime values for CdTe can often be problematic, however, owing to enhanced surface recombination^17^. This may be overcome by techniques such as two photon excitation measurements^18^, but here we instead employ two different single photon measurement protocols: one where excitation occurs through the glass, called ‘front surface'; and one where illumination occurs at the free CdTe back surface. Figure 4 shows both front and back surface TRPL measurements from a 10 μW 760 nm excitation (5 μm diameter spot) for (a) MgCl_2_, (b) CdCl_2_, (c) NaCl and (d) KCl-treated cells, while the average carrier lifetime values extracted are given in Fig. 4e (the same decay curves are plotted in log scale in Supplementary Fig. 4 for reference). For back surface measurements, the minority-carrier lifetimes are, as expected, dominated by surface recombination. Hence the lifetimes for all chlorides do not deviate from the as-grown value, 0.24 ns, by more than the experimental error. Back surface measurement is, therefore, considered to yield surface-dominated lifetime values that are not representative of the bulk carrier lifetime. In the case of front surface measurements, however, there is a far greater variation in the determined lifetime values. For the as-grown sample the lifetime is 0.30±0.04 ns but following successful chloride treatments, using CdCl_2_ or MgCl_2_, this lifetime is increased to >1.6 ns, which correlates with the increased in *V*_OC_ seen in these cells (>0.8 V from 0.650 V). For NaCl and KCl treatments, the carrier lifetime is marginally reduced compared with the as-grown sample to 0.25±0.01 and 0.28±0.07 ns, respectively, but remains within bounds of error. Again these results are consistent with the similar *V*_OC_ levels for these treatments.

These results, in conjunction with the EELS data, suggest that the increased carrier lifetimes, and thus voltages, associated with CdCl_2_ and MgCl_2_ treatments are primarily due to reduced recombination at grain boundaries following chlorine inclusion in these regions. On the contrary, the largely unchanged carrier lifetimes for NaCl and KCl treatments indicate why—despite the increase in efficiency—they are unable to increase *V*_OC_. For such treatments, the grain boundaries are not passivated and recombination persists at similar levels to that in as-grown samples, which demonstrates that cell *V*_OC_ is essentially grain boundary dominated. Nevertheless, efficiency improvement still occurs via increases in *J*_SC_ (<15 mA cm^−2^ as grown, to >20 mA cm^−2^ for NaCl treatment) and fill factor (<32% as grown, to >56% for NaCl treatment), which importantly occur even in the ‘ineffective' chlorides (or indeed air annealing, Supplementary Fig. 3) even though insufficient chlorine is introduced to change *V*_OC_. It seems obvious then that these improvement occur via a distinctly separate mechanism, which is primarily related to the annealing process rather than chlorine incorporation. This process may be investigated by studies of the junction position and recombination mechanisms.

### Electron beam-induced current (EBIC) analysis

**Figure 5:**
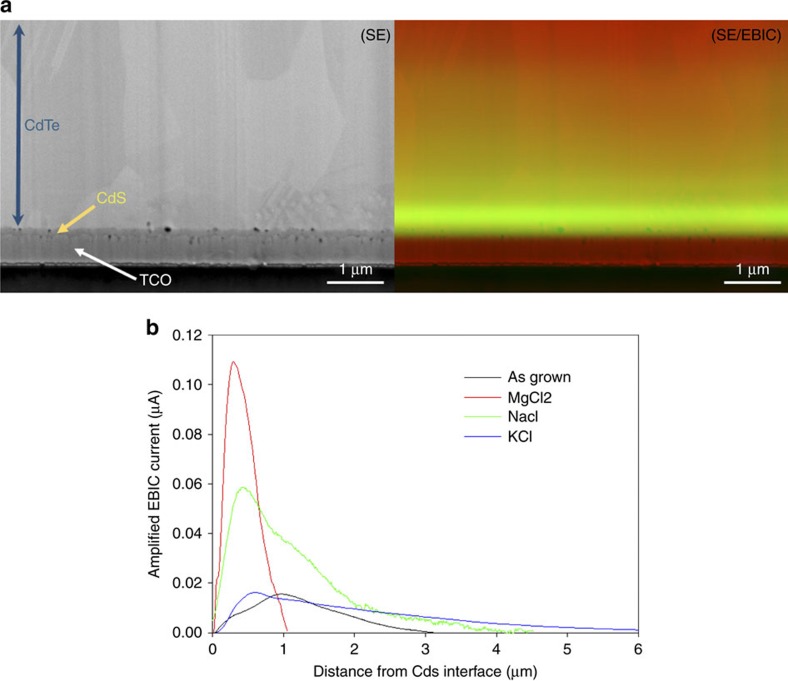
EBIC analysis of cell cross sections. (**a**) Secondary electron (SE), EBIC and combined SE/EBIC images for an NaCl-treated solar cell; (**b**) quantitative EBIC current data as a function of beam position relative to the CdS/CdTe interface for as-grown (black), MgCl_2_ (red), NaCl (green) and KCl (blue)-treated cells. All measurements are shown on the same distance time scale to allow direct comparison, traces end where the EBIC current drops below the detector threshold.

Cross sectional EBIC analysis is an SEM-based technique where the current response of a cell is monitored in relation to the position of the electron beam. For CdTe solar cells, this allows determination of the photovoltaic junction position within the cell structure to a high degree of accuracy^7^,^19^,^20^. CdTe cells treated with MgCl_2_, KCl NaCl, and an as-grown sample were compared. Figure 5a shows a typical EBIC micrograph for an NaCl-treated cell, with those for the other samples being available in Supplementary Fig. 5. Figure 5b gives the quantitative linescans of the amplified EBIC current value as a function of distance from the CdS interface for all cells analysed. Since the amplifier settings were identical in all cases, the curves may be compared directly: it may be seen that all of the chloride treatments have an effect on the position of the photovoltaic junction compared with the as-grown case, and move it towards the CdS interface.

The EBIC analysis shows that all treatments have an effect on the location of the photovoltaic junction. For the as-grown sample the cell has a buried junction^21^ (n-CdTe/p-CdTe) centred at ∼960 nm from the TCO/CdS interface. It has been suggested that apparent shifts in junction position observed by EBIC may potentially result from high-injection beam conditions^22^ (that is, where the injected electron density vastly exceeds the materials doping density). In these instances the junction is, to an extent, deactivated and collection is instead controlled by the back contact field, with the peak of EBIC collection, therefore, occurring away from the interface and close to the back contact. For the measurements reported here, there is no evidence of high injection effects as collection still occurred comparatively close to the CdS/CdTe interface. The shift in junction position for the as-grown sample can additionally be corroborated via both external quantum efficiency (Supplementary Fig. 6), which shows characteristic buried junction behaviour, and capacitance voltage profiling (Supplementary Fig. 7), which shows the junction position deeper into the CdTe. Both of these measurements are obviously independent of injection effects. Hence, we are satisfied that any EBIC peak shifts observed are a material property rather than being due to high-injection effects.

Following MgCl_2_ treatment the junction is brought close to the interface, (as seen for CdCl_2_ treatment^20^,^23^), and the peak current is increased by approximately an order of magnitude relative to the as-grown case. Intermediate behaviour is seen for the ineffective chlorides—the junction is brought closer to the interface (NaCl ∼400 nm, KCl ∼590 nm), and the current peaks are increased—but the effects are much weaker for KCl than for NaCl, and neither is as effective as MgCl_2_. It is this shift in junction position that results in the improved *J*_SC_ and fill factor for all the treated devices over the as-grown ones, but the shallow junction position resulting from MgCl_2_ and CdCl_2_ are the most effective.

While the MgCl_2_ treatment, similarly to the CdCl_2_ treatment^20^, shows the expected junction position located at the CdS/CdTe interface, it is interesting to note the shift in junction position for NaCl and KCl treatments. These shifts observed via EBIC may be caused by either a modification in the recombination rate at the near interface region^24^, pushing the observed EBIC collection peak deeper into the CdTe, or else by some change in the n- and/or p-carrier concentration levels. In the case of NaCl and KCl treatments whatever the mechanism it has occurred despite the lack of chlorine incorporation. Oxygen has been reported to act a p-type dopant in CdTe^25^,^26^, which may feasibly lead to type conversion, however, SIMS analysis showed little increase in the oxygen content of the films in this case (Supplementary Fig. 8). It would seem reduced recombination at the interface may offer a better explanation, however, EBIC measurements alone are insufficient to separate the effects of type conversion from alteration of the junction recombination. By measurement and analysis of JV curves as a function of temperature (*JVT* analysis) though, it is possible to assess the electrical transport and recombination occurring in the p–n junction.

### Carrier transport analysis

**Figure 6:**
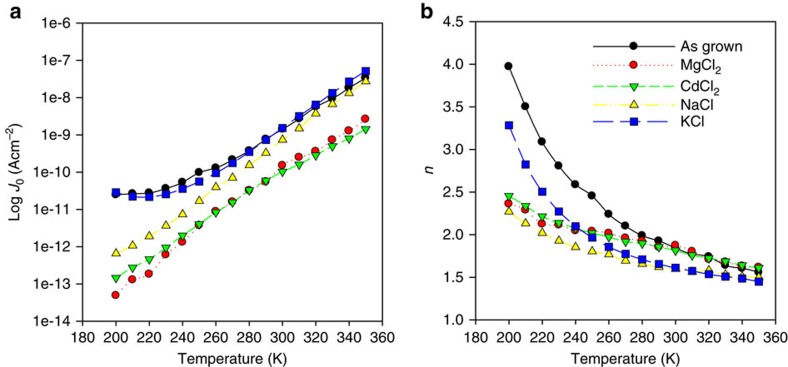
JVT analysis. Values extracted as a function of temperature from *JVT* analysis of as-grown (black), MgCl_2_ (red) CdCl_2_ (green), NaCl (yellow) and KCl (blue)-treated cells (**a**) the saturation current *J*_0_ and (**b**) *n* the ideality factor.

Analysis of *JVT* data for as-grown, CdCl_2_, MgCl_2_, KCl and NaCl-treated devices was performed via the slope method (see methods section and Supplementary Fig. 9), and used to determine both the ideality factor *n* and the reverse saturation current, *J*_0_, which are plotted as a function of temperature in Fig. 6a,b. These quantities are good markers for the device junction quality and level of recombination at the interface. The recombination current occurs due to a flow of carriers recombining in the depletion region and may take place via either direct band-to-band recombination or indirect trapping assisted recombination routes^27^. Trap assisted transport mechanisms are commonly observed in CdTe solar cells due to a high degree lattice mismatch between CdS/CdTe layers, leading to the formation of interfacial defects^28^. Although numerous transport mechanisms have been reported for CdTe solar cells, the two most common are trap dominated multi-step tunnelling^29^ and Shockley-Read-Hall (SRH) recombination^30^. For device systems where the SRH model breaks down and carrier transport in the junction is dominated by trap assisted recombination, we would anticipate higher values of *J*_0_ and values of *n*>2. Therefore, for higher quality junctions we would anticipate a reduction in the value of both *J*_0_ and *n*. It can be seen from Fig. 6a that the *J*_0_ values determined for MgCl_2_ and CdCl_2_ treatments are significantly reduced at all temperatures compared with the as-grown device, while NaCl treatment provides a smaller decrease and the KCl-induced reduction is negligible compared with the as-grown device.

At low temperatures (<240 K) KCl and as-grown samples also displayed values of *n* much higher than for MgCl_2_, CdCl_2_ and NaCl treatments, with all values being >2. For NaCl and KCl treatments, it would therefore appear that there are still a greater number of defects acting as recombination centres, than for MgCl_2_ and CdCl_2_. The improvement in junction quality, for MgCl_2_ andCdCl_2_ treatments seems likely to be due a combination of chlorine incorporation at grain boundaries and reduced strain at the CdS/CdTe interface. For NaCl and KCl processing any changes occur primarily as a results of thermal annealing and thus reduced strain, due to the lack of chlorine incorporation detected by SIMS. Hence, it seems likely that for these devices carrier transport at the junction is dominated by recombination at defect states and that the chloride process has a significant role in reducing this recombination. Due to the preferential incorporation of chlorine at the grain boundaries detected by EELS analysis, it would appear these defect sites primarily reside at the grain boundaries. For treatments where chlorine is inadequately incorporated we see a slight reduction in the recombination current, but that there is still significant recombination in the near interface region due to the higher grain boundary population in this region. Hence, MgCl_2_ and CdCl_2_ are seen to create higher quality junctions than the other chlorides due to effective passivation of these defect states. The *JVT* analysis is in good agreement with EBIC position being shifted deeper into the junction for cells with higher recombination that is, recombination at the near interface has forced the EBIC observed junction deeper into the material. Combining *JVT* and EBIC analysis, the obvious conclusion is that reduced interfacial recombination causes the shift in the effective junction position, which in turn leads to improvements in cell *J*_SC_ and fill factor values.

## Discussion

These results demonstrate that what is commonly thought of as a single-chloride process may actually be an effect of two overlapping process. Device performance may be improved through increases in *J*_*SC*_ and fill factor when little or no chloride is contributed to the cell as a result of annealing. This serves to partially reduce recombination in the near interface region and move the junction towards the CdS interface. The key role of chlorine within the cells is to increase *V*_OC_ through passivation of the CdTe grain boundaries and thereby generate a subsequent increase in the carrier lifetime. Without this the cell *V*_OC_ does not improve, a finding which is of relevance to other thin film technologies such as CZTS and CIGS, which have similar grain structure and similarly limited device *V*_OC_ relative to their theoretical maximum. These results indicate that the key route to improving the cell open circuit voltage, the performance-limiting factor for CdTe solar cells, could, therefore, be through focussing on improving the grain boundary passivation. This may potentially be achieved by separating the annealing and chloride in-diffusion steps that is, by air annealing first followed by a separate chlorine in-diffusion step. In the present methodology, two processes are being combined, but by first annealing the solar cell and then attempting grain boundary passivation separately, the in-diffusion of chlorine may be more accurately optimized or even replaced by an alternative. It may be that MgCl_2_ and CdCl_2_ are effective simply because they dissociate chlorine in the correct temperature range for annealing. If the annealing step can be separated lower temperature or more controllable chlorine processing may be possible. By demonstrating that both MgCl_2_ and CdCl_2_ are simply sources of chlorine in-diffusion rather than the compounds themselves being in some way essential, we hope this may inform development of additional non-toxic chloride activation routes and enable industrial fabrication processes to move away from their reliance on hazardous CdCl_2_ process. Industrial manufacturers are often naturally conservative with regards to changing their processes, but a more developed understanding of the activation mechanism will hopefully help provide the confidence to accept a more environmentally safe procedure.

## Methods

### Cell deposition and processing

All solar cells were deposited in the superstrate geometry using SnO_2_:F (FTO) coated soda-lime TEC glass, supplied by NSG Ltd. A 100 nm thick ZnO buffer layers was deposited by sputtering at room temperature followed by 120 nm thick CdS at 200 °C. Close space sublimation was used to deposit CdTe layers. Source and substrate temperatures were 605 and 520 °C, respectively, with a nitrogen growth ambient at a pressure of 25 Torr. All samples were etched for 30 s in a nitric-phosphoric acid etch solution following CdTe deposition but before chloride treatment^31^. A second 30 s nitric-phosphoric etching step was applied following chloride treatment to both clean the surface and create a Te-rich layer before contacting. Back contacting was via 0.25 cm^2^ gold contacts deposited by thermal evaporation. No copper was intentionally included in cell deposition or back contacting.

For comparison as-grown (that is, no chloride treatment) and CdCl_2_-treated samples were also prepared. The as-grown sample was not subjected to any post-growth annealing but was nitric-phosphoric etched for 60 s to match the other samples. The CdCl_2_ layer was 100 nm thick and deposited by thermal evaporation. Other chlorides were deposited from a solution of 10% chloride to 90% methanol by weight. Samples were coated with a few drops of solution on the CdTe back surface before annealing. All treated samples were annealed in a tube furnace in an air ambient, with treatment conditions being optimized in the range 410–450 °C and 10–60 min for each chloride. Comparative air annealing samples were annealed in a separate furnace, which had not been used for any chloride annealing process to avoid any chlorine inclusion.

### Electrical characterization

All current voltage data was recorded under AM1.5 spectrum using a TS Space Systems solar simulator at 1,000 Wm^−2^ determined using a calibrated GaAs photodiode. Gold back contacts had an area of 0.25 cm^2^ defined by scribing. As an aperture was not used for *JV* measurements, an area definition error of 0.02 cm^2^ was assumed to account for variations in contact size, however, contact preparation and measurement conditions were identical in each case. Measurements were performed without any form of voltage or light soaking before measurement. Measurements were made from −1 V to +1 V and no hysteresis effects were observed as forward and reverse measurements produced identical data. Example external quantum efficiency (EQE) data is included in Supplementary Fig. 6. EQE measurements were calibrated using a Si photodiode and recorded in the dark, that is, without an AM1.5 white light bias. The lack of AM1.5 biasing may lead to some variation in the under-curve area in comparison to *J*_SC_ values, particularly for lower performance cells (for example, as-grown, KCl treated), owing to a variation in the population of defect states and thus recombination at low light intensities^32^,^33^,^34^. *JVT* measurements were made using a Keithley 2,400 source-meter and a CTI-cryogenics cryostat over a temperature range of 150–350 K. *C*–*V* measurements were performed at a frequency of 1 kHz using a Solatron SI1260 impedance analyser in dark conditions.

### Electron energy loss spectrometry

EELS analysis was carried out using a fifth order aberration corrected Nion UltraSTEM 200 equipped with a Gatan Enfinium EELS Spectrometer. Measurements were made using an accelerating voltage of 200 kV and a 30 pA beam current. Sample thinning was done by mechanical polishing, followed by argon ion milling in liquid nitrogen temperature. Cl L_23_, Cd M_45_, and Te M_45_ edges were used for the EELS elemental maps with a collection angle of ∼36 mrad.

### Time-resolved photoluminescence

Time-resolved measurements were performed using a time correlated single-photon counting system with a temporal resolution of 45 ps. All samples were excited with 10 μW power at 760 nm with 120 fs pulses at 800 kHz repetition rate focused onto the sample with a 40 × objective to a 5 μm spot diameter.

The decay kinetics were found to show a bi-exponential decay profile described by the function:


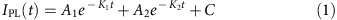


with the average carrier lifetime given by;


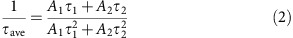


where *τ*_1_=1/*k*_1_ and *τ*_2_=1/*k*_2_.

### Electron beam-induced current

Samples were prepared for EBIC analysis using focussed ion beam (FIB) cross-section milling^35^ with a Ga liquid metal ion source FEI Helios Nano Lab 600 Dual Beam system. A series of *in situ* polishing steps were performed to produce a clean surface with minimal beam damage. Simultaneous EBIC analysis and secondary electron imaging was carried out in a Hitachi SU-70 microscope with EBIC signals being collected through a Matelect ISM6 specimen current amplifier, using a beam voltage of 5 kV and current of 0.75 nA. The image filter frequency was set to 10 kHz—this eliminated both noise and fine image detail, and so the images in this paper show the average position of the junctions in the PV devices.

### Current–voltage–temperature (*JVT*) analysis methodology

From the Shockley equation, ln*J* for a forward-biased p*–*n junction must vary linearly with *V*. However, deviation from the expected straight line is usually observed for real devices. Supplementary Fig. 9 shows a typical ln*J* versus *V* curve for JV data recorded for a MgCl_2_-treated cell at 350 K. Curves usually display three distinctive regions; low bias, linear region and high bias. At high forward bias, region III, series resistance effects are dominant and limit the current flow, often leading to the phenomenon of rollover^36^. In the low bias region I, deviation occurs due to several factors such as (i) shunting, and (ii) high turn-on voltage, which increases as saturation current decreases. The part of the *JV* curve for *V* less than *V*_turn-on_ is non-exponential, and thus produces the distortion in the curve observed in region I. Region II is the linear portion of the curve, which represents the expected behaviour of the junction, having negligible effects of the parasitic resistances (series and shunt resistances). Thus, data from region II is used for parameter extraction, since here the Shockley equation is valid. At low temperature, the factors causing the deviation of the ln*J* versus *V* curve in regions I and III are more pronounced. Thus, these regions expand at the expense of region II, a limitation that may induce errors in parameter extraction. Therefore, in this work despite *JV* data being recorded over a temperature range of 150–350 K only data in the range of 200–350 K was considered.

For analysis of *JV* curves as a function of temperature the slope method was used to extract data^37^, whereby a linear fit is made to the data for region II. From this we may determine the gradient of the fit, *A*, from which the ideality factor *n* is calculated. The reverse saturation current, *J*_0_, is determined from the *y*axis intercept. From the response of the *A, J*_0_ and *n* to temperature the dominant mechanism may be inferred.

For multi-step tunnelling dominated carrier transport, whereby carriers recombine via a staircase of trapping states distributed throughout the energy gap, the current density passing through the junction under forward bias obeys the following relationship:


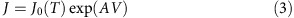


Hence, for junctions where multi-step tunnelling dominates *A* should be temperature independent while ln*J*_0_ should show an inverse linear temperature dependence. For a multi-step tunnelling regime, we also expect that *n* should decrease for increasing temperature while for some other transport mechanisms (for example, SRH) it should be temperature independent. For example as-grown and KCl devices examined show some deviation from this linear relationship at low temperature (<250 K), however, this is most likely caused by the effect of particularly high parasitic resistances present in these devices. This may be confirmed by the temperature dependence of the ideality factor, which decreases with increasing temperature for carrier transport dominated by multi-step tunnelling.

In the case of multi-step tunnelling, it is possible to determine the number of tunnelling steps (*R*) for carriers traversing the depletion region from the value of *A* by using the following equation:


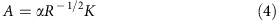


where,


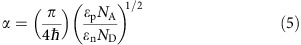



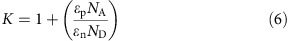


and *m*_n_ is the electron effective mass, *ɛ*_n_ and *ɛ*_p_ are the dielectric constants of the n- and the p-side of the junction, respectively. *N*_A_ and *N*_D_ are the acceptor and donor doping concentrations, respectively. A reduction in the number of tunnelling steps is generally considered to reflect a reduction in the number of interface states and an improvement in the junction quality. Equations (4, 5, 6) were used, in conjunction with carrier concentrations extracted from capacitance-voltage measurements, to calculate the number of tunnelling steps required for the carriers to pass through the depletion region. It is well known that the doping concentration of CdS is a few orders of magnitude higher than that for CdTe and thus the approximation *K*≈1 (see equation (6)) is used^38^,^39^. By using the values of CdTe doping concentration, the parameter *α* is calculated from equation (5) (using the typical values of the electron effective mass and the dielectric constants of CdTe). Finally, substituting these values in equation (4) yields the number of tunnelling steps *R*. Note that the parameter *A* is supposed to be constant throughout the whole range of *T*. However, due to its temperature variation, the value at room temperature was used in the calculation for all samples.

### Data availability

The data which supports the findings of this work is available through the University of Liverpool RDM DataStore or from the author by request.

## Additional information

**How to cite this article:** Major, J. D. *et al*. In-depth analysis of chloride treatments for thin-film CdTe solar cells. *Nat. Commun.* **7,** 13231 doi: 10.1038/ncomms13231 (2016).

## Supplementary Material

Supplementary Information Supplementary Figures 1-9, Supplementary Table 1 and Supplementary References.
