# Supplementary material for: In-depth analysis of chloride treatments for thin-film CdTe solar cells
Source: Nat Commun. 2016 Oct 24;7:13231. doi: 10.1038/ncomms13231 (PMC5078995; doi:10.1038/ncomms13231)
Supplement: Supplementary Information — Supplementary Figures 1-9, Supplementary Table 1 and Supplementary References. [file ncomms13231-s1.pdf]

Supplementary Information

Supplementary Figures

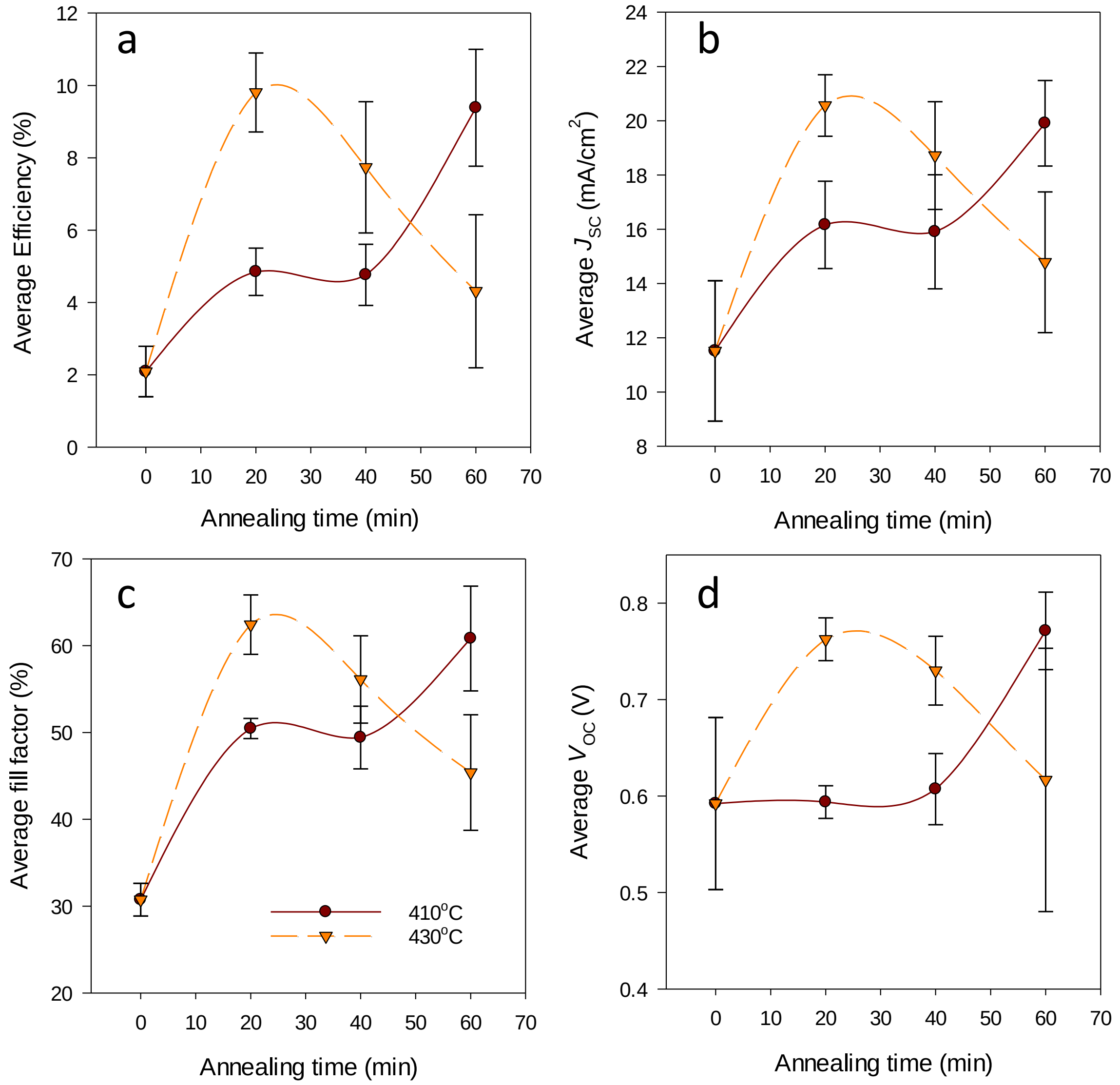

Supplementary Figure 1 Cell performance for $MgCl_2$ treatment: Variation in average performance parameters extracted from *JV* data for $MgCl_2$ treatment performed for a range of treatment times and temperatures 410°C (red) and 430°C (orange), a) efficiency b) short circuit current density, c) fill factor and d) open circuit voltage. Each data point is an average of 9 contacts with error bars being the standard deviation and an error term for contact size variation.

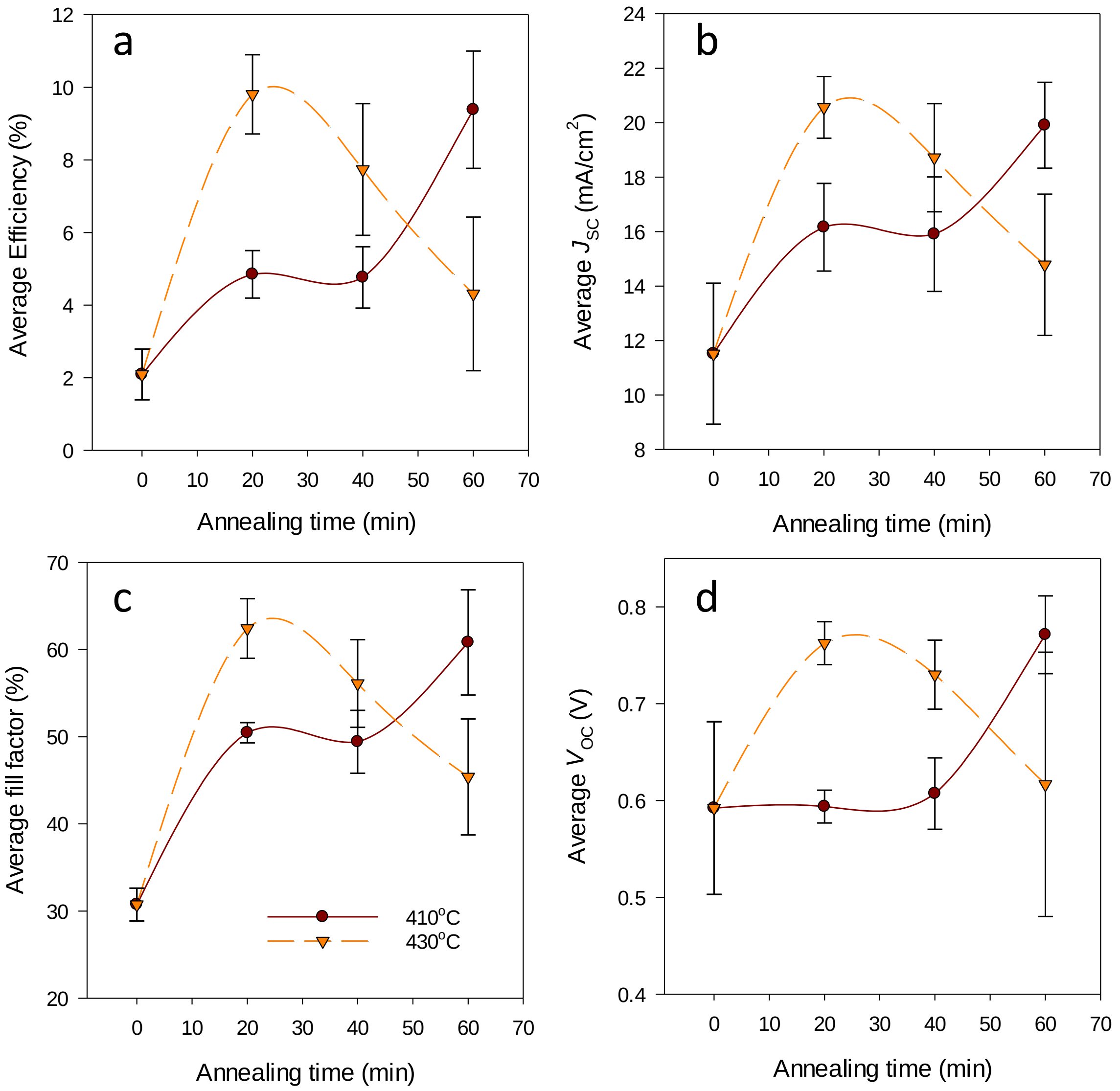

Supplementary Figure 2 Cell performance for NaCl treatment: Variation in average performance parameters extracted from *JV* data for NaCl treatment performed for a range of treatment times and temperatures 410°C (red), 430°C (orange) and 450°C (yellow), a) efficiency b) short circuit current density, c) fill factor and d) open circuit voltage. Each data point is an average of 9 contacts with error bars being the standard deviation and an error term for contact size variation.

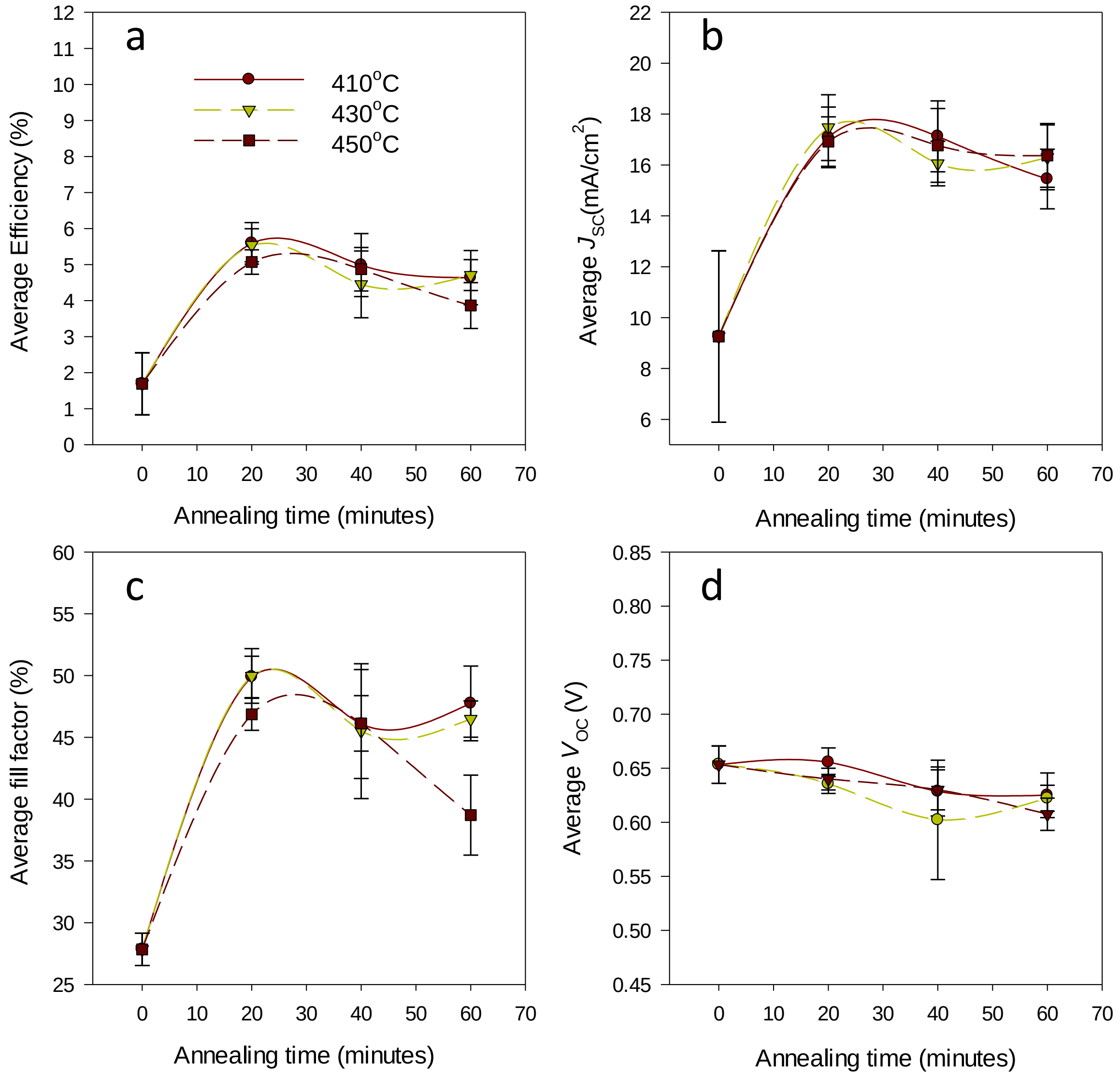

Supplementary Figure 3 Cell performance for air annealing: Variation in average performance parameters extracted from *JV* data for air annealed cells at a range of treatment times and temperatures 410°C (red), 430°C (yellow) and 450°C (orange), a) efficiency b) short circuit current density, c) fill factor and d) open circuit voltage. Each data point is an average of 9 contacts with error bars being the standard deviation and an error term for contact size variation.

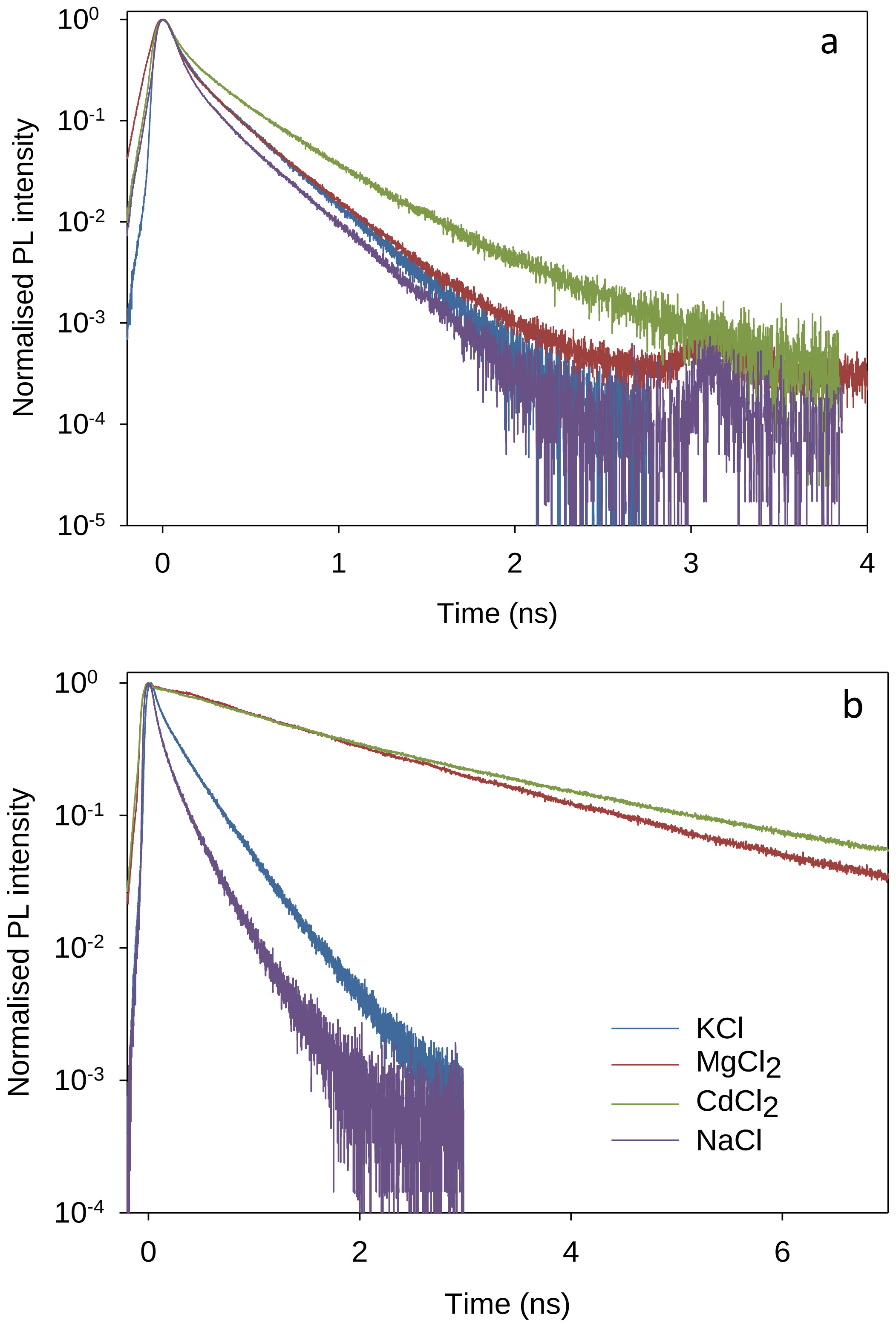

Supplementary Figure 4 Log scale TRPL data: Log scale versions of TRPL decay curves for KCl (blue), $MgCl_2$ (red), $CdCl_2$ (green) and NaCl (purple) given in figure 4 for a) back surface and b) front surface excitation.

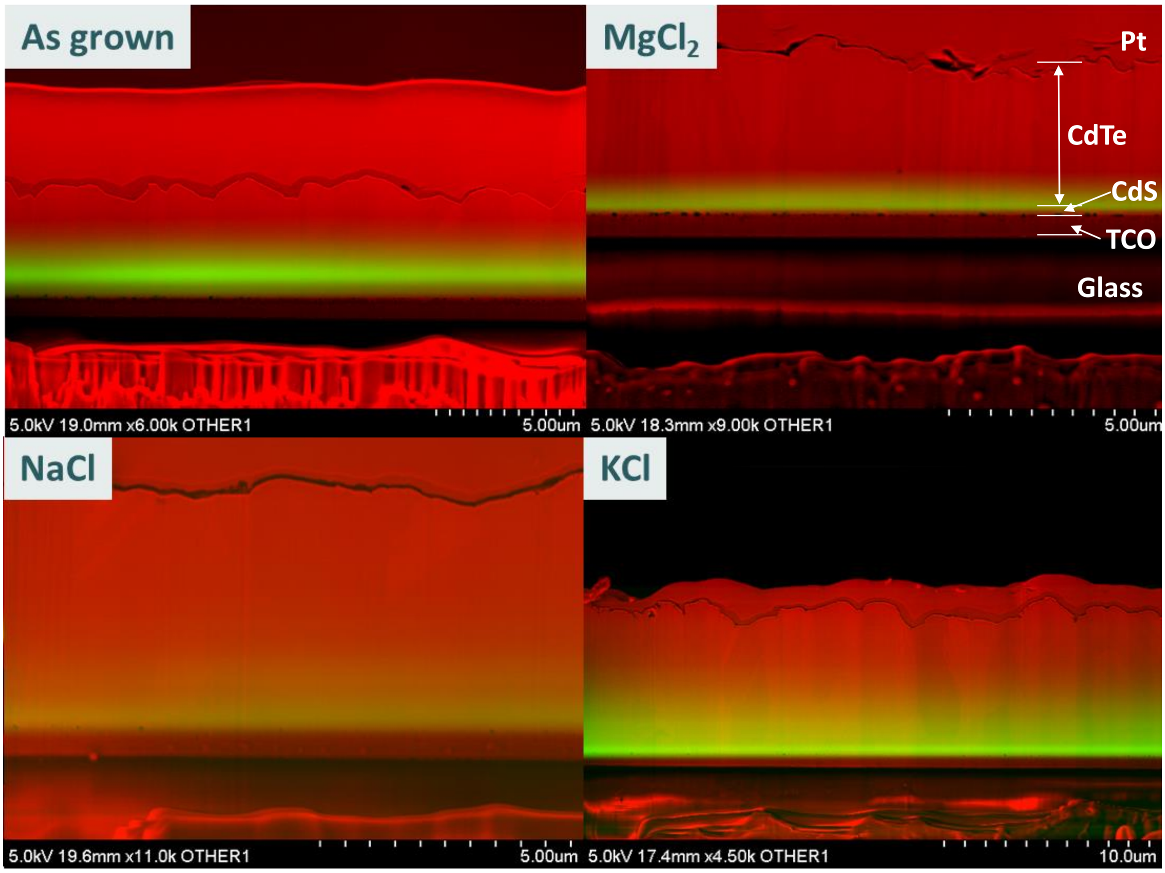

Supplementary Figure 5 Cross sectional EBIC analysis: EBIC current signal (green) overlaid on the secondary electron image (red) for as-grown, $MgCl_2$, NaCl and KCl treated CdTe solar cells.

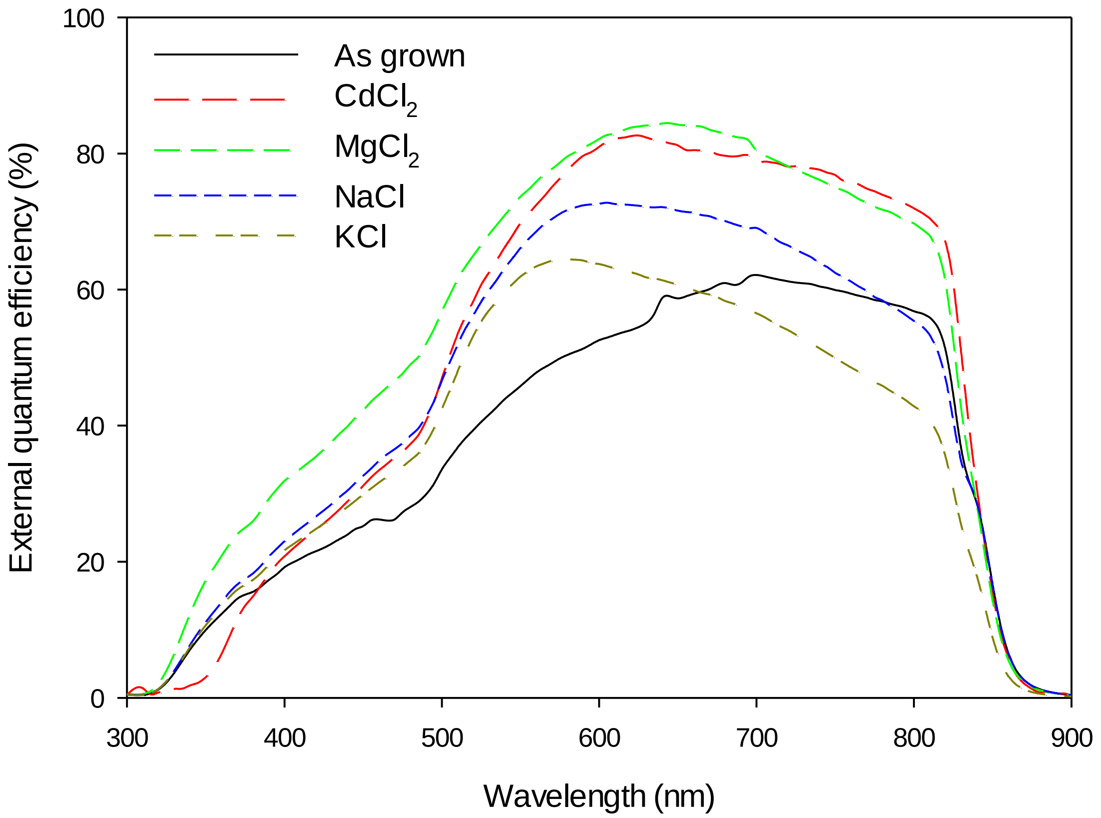

Supplementary Figure 6 External quantum efficiency: External quantum efficiency measurements for typical contacts from cells treated with each of the compared chlorides. Measurements were recorded under dark conditions (*i.e.* no white light bias) using a Bentham PVE300 system.

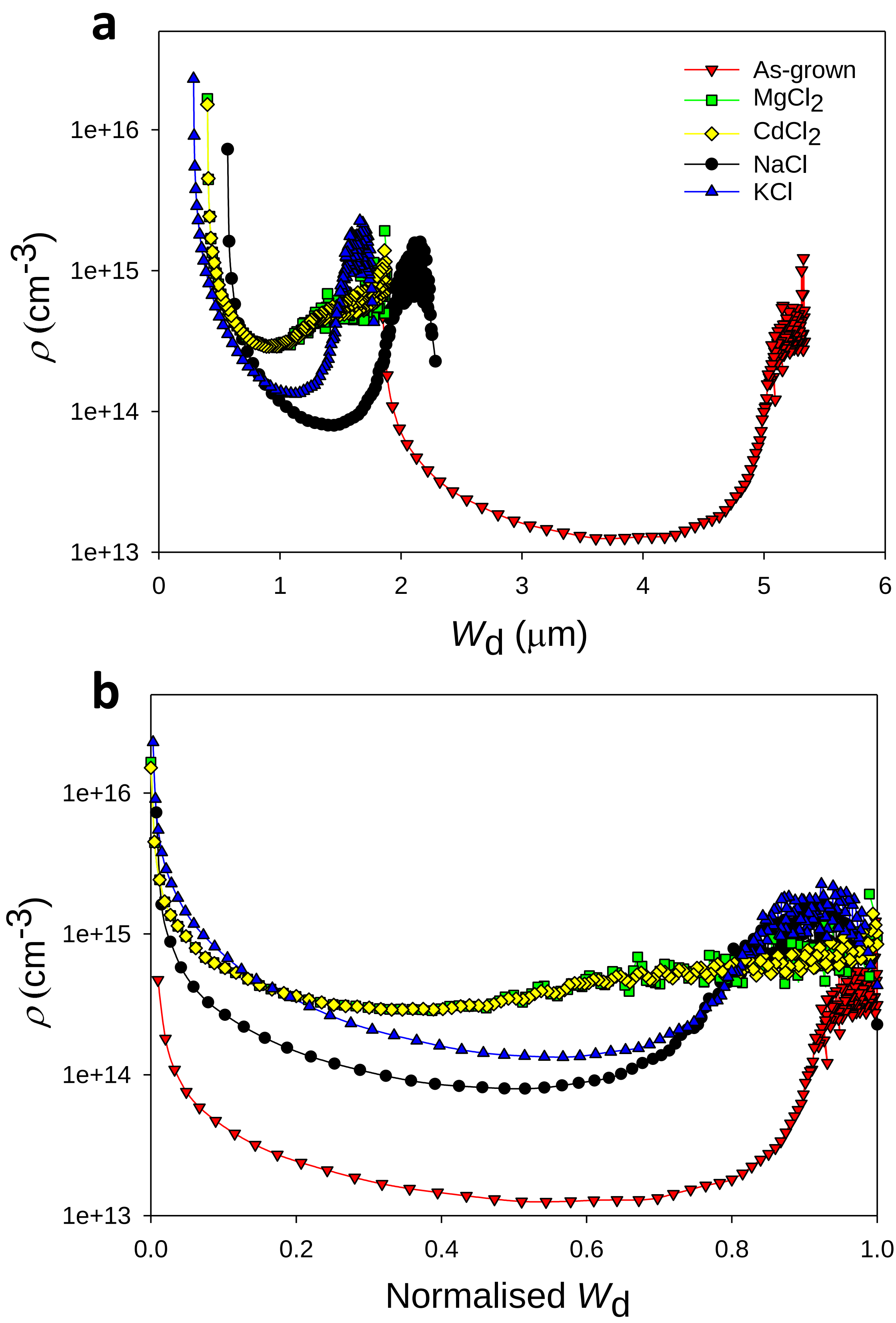

Supplementary Figure 7 Capacitance voltage analysis: Hole density (ρ) versus a) depletion width $W_d = \varepsilon\varepsilon_0 A/C$ and b) normalised depletion width, determined from capacitance voltage (C-V) measurements for different chloride treatments. W_d=0μm corresponds to the CdS/CdTe interface. Only V_{bias} <500mV are included due to back contact capacitive effects[3].

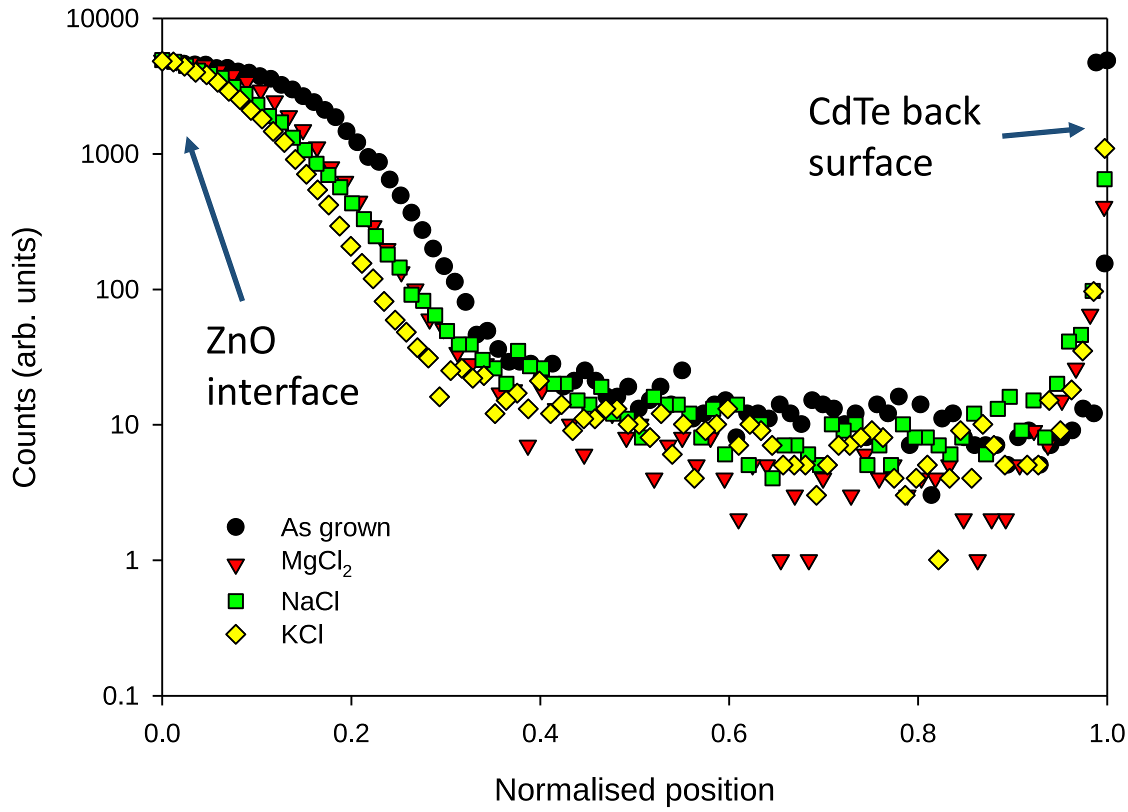

Supplementary Figure 8 SIMS oxygen profiles: SIMS profiles of oxygen content in as-grown (black), $MgCl_2$ (red), KCl (yellow) and NaCl (green) treated CdTe solar cells.

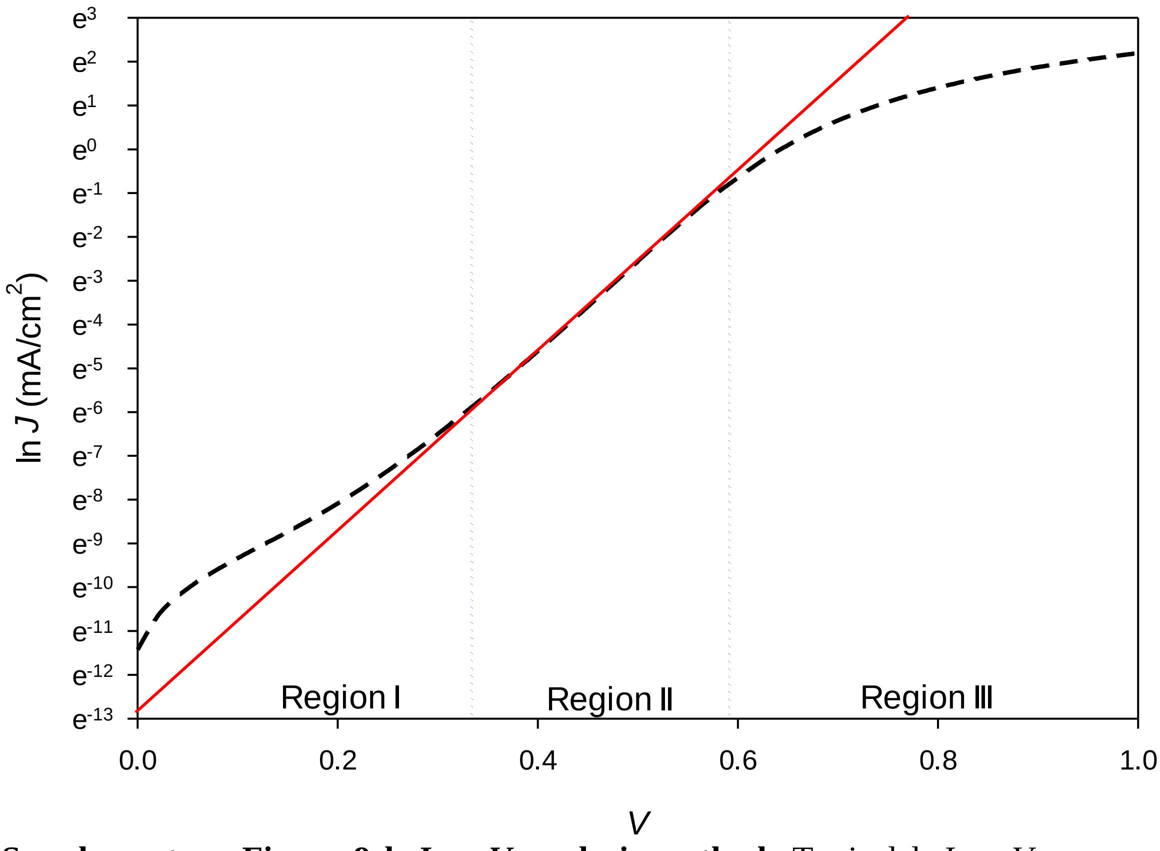

Supplementary Figure 9 ln*J* vs *V* analysis method: Typical ln*J* vs *V* response at forward bias for a p-n junction. The figure shows the deviation from linear response at low (region I) and high (region III) forward bias. A linear fit (red) was used to determine J_0, *n* and *A* values.

Supplementary Tables

Treatment	Peak efficiency (%)	Peak fill factor (%)	Peak J_{SC} (mAcm^{-2})	Peak V_{OC} (V)
**$CdCl_2$**	**13.02**	**70.01**	**22.13**	**0.831**
**$MgCl_2$**	**12.71**	**69.08**	**22.41**	**0.821**
**NH_4Cl**	**10.31**	**63.29**	**22.02**	**0.747**
**HCl**	**8.75**	**60.98**	**21.42**	**0.611**
**NaCl**	**7.57**	**56.26**	**20.08**	**0.670**
**KCl**	**5.49**	**50.11**	**17.95**	**0.607**
**$MnCl_2$**	**4.37**	**45.87**	**18.30**	**0.520**
**As-grown**	**2.99**	**31.77**	**14.51**	**0.650**

Supplementary Table 1: Summarised cell parameters: Comparison of peak performance parameters for equivalent cells with different treatment from Major et al[1] and for NH_4Cl treatment[2]. Data is from in-house non-aperture defined contacts. Contact preparation and measurement conditions were identical in each case.

Supplementary References

1. J.D. Major, R.E. Treharne, L.J. Phillips, K. Durose, A low-cost non-toxic post-growth activation step for CdTe solar cells, Nature, **511**, 334-337 (2014).
2. J.D. Major, L. Bowen, R.E. Treharne, L.J. Phillips, K. Durose, NH4Cl alternative to the CdCl2 Treatment Step for CdTe Thin Film Solar Cells, IEEE Journal of Photovoltaics., **5**, 386-389 (2015).
3. J.V. Li, A.F. Halverson, O.V. Sulima, S. Bansal, J.M. Burst, T.M. Barnes, T.A. Gessert, D.H. Levi, Theoretical analysis of effects of deep level, back contact, and absorber thickness on capacitance-voltage profiling of CdTe thin-film solar cells, Solar Energy Materials and Solar Cells, **100**, 126-131 (2012).
